# Low β_2_-adrenergic receptor level may promote development of castration resistant prostate cancer and altered steroid metabolism

**DOI:** 10.18632/oncotarget.6479

**Published:** 2015-12-04

**Authors:** Peder Rustøen Braadland, Helene Hartvedt Grytli, Håkon Ramberg, Betina Katz, Ralf Kellman, Louis Gauthier-Landry, Ladan Fazli, Kurt Allen Krobert, Wanzhong Wang, Finn Olav Levy, Anders Bjartell, Viktor Berge, Paul S. Rennie, Gunnar Mellgren, Gunhild Mari Mælandsmo, Aud Svindland, Olivier Barbier, Kristin Austlid Taskén

**Affiliations:** ^1^ Department of Tumor Biology, Institute for Cancer Research, Oslo University Hospital, Oslo, Norway; ^2^ Department of Pathology, Oslo University Hospital, Oslo, Norway; ^3^ Hormone Laboratory, Haukeland University Hospital, Bergen, Norway; ^4^ Laboratory of Molecular Pharmacology, CHU-Québec Research Center and Faculty of Pharmacy, Laval University, Québec, Canada; ^5^ The Vancouver Prostate Centre, University of British Columbia, Vancouver, Canada; ^6^ Department of Pharmacology, Institute of Clinical Medicine, University of Oslo and Oslo University Hospital, Oslo, Norway; ^7^ K.G. Jebsen Cardiac Research Centre and Center for Heart Failure Research, Faculty of Medicine, University of Oslo, Oslo, Norway; ^8^ Department of Medical Biosciences, Pathology, Umeå University, Umeå, Sweden; ^9^ Department of Urology, Skåne University Hospital, Malmø, Sweden; ^10^ Department of Clinical Sciences Malmø, Division of Urological Cancers, Lund University, Lund, Sweden; ^11^ Department of Urology, Oslo University Hospital, Oslo, Norway; ^12^ Department of Clinical Science, University of Bergen, Bergen, Norway; ^13^ Institute for Pharmacy, Faculty of Health Science, University of Tromsø, Tromsø, Norway; ^14^ Institute of Clinical Medicine, University of Oslo, Oslo, Norway

**Keywords:** β2-adrenergic receptor, ADRB2, CRPC, UGT2B15, UGT2B17

## Abstract

The underlying mechanisms responsible for the development of castration-resistant prostate cancer (CRPC) in patients who have undergone androgen deprivation therapy are not fully understood. This is the first study to address whether β_2_-adrenergic receptor (ADRB2)- mediated signaling may affect CRPC progression *in vivo.* By immunohistochemical analyses, we observed that low levels of ADRB2 is associated with a more rapid development of CRPC in a Norwegian patient cohort. To elucidate mechanisms by which ADRB2 may affect CRPC development, we stably transfected LNCaP cells with shRNAs to mimic low and high expression of ADRB2. Two UDP-glucuronosyltransferases, UGT2B15 and UGT2B17, involved in phase II metabolism of androgens, were strongly downregulated in two LNCaP shADRB2 cell lines. The low-ADRB2 LNCaP cell lines displayed lowered glucuronidation activities towards androgens than high-ADRB2 cells. Furthermore, increased levels of testosterone and enhanced androgen responsiveness were observed in LNCaP cells expressing low level of ADRB2. Interestingly, these cells grew faster than high-ADRB2 LNCaP cells, and sustained their low glucuronidation activity in castrated NOD/SCID mice. ADRB2 immunohistochemical staining intensity correlated with UGT2B15 staining intensity in independent TMA studies and with UGT2B17 in one TMA study. Similar to ADRB2, we show that low levels of UGT2B15 are associated with a more rapid CRPC progression. We propose a novel mechanism by which ADRB2 may affect the development of CRPC through downregulation of UGT2B15 and UGT2B17.

## INTRODUCTION

Androgen deprivation therapy (ADT) is the first line of treatment for patients with advanced or metastatic prostate cancer [1]. ADT is initially effective in controlling tumor growth and symptoms, but most tumors eventually develop resistance to ADT and become castration resistant prostate cancers (CRPC). Over the last years, it has become evident that the androgen signaling axis plays a pivotal role in the development of CRPC [2]. The multiple molecular mechanisms by which the androgen receptor (AR) contributes to disease progression despite castration levels of androgens in prostate cancer have been thoroughly reviewed [3-6]. Several new targets in the AR activation pathway have emerged in recent years [7, 8]. The steroidogenic pathway has received increasing attention, as drugs targeting this pathway, such as abiraterone (an inhibitor of cytochrome P450, family 17, subfamily A, polypeptide 1 (CYP17)) improve the life expectancy of patients with CRPC, despite the assumed androgen-independence of these cancer cases [8]. No curative options for CRPC are, however, available today. Increased knowledge of the mechanisms by which the cancer cells progress to CRPC is hence needed. Recently, targeting the androgen extrahepatic phase-II metabolic pathways has arisen as a potential tool to help maintain androgen-deprived conditions during ADT [9]. The UDP-glucuronosyltransferases 2B15 (UGT2B15) and 2B17 (UGT2B17) are of special interest, as they are expressed in prostate tissue and cell lines, and they exhibit specificity for androgen metabolites [10].

The β_2_-adrenergic receptor (ADRB2) and its downstream effectors cyclic AMP (cAMP) and cAMP-dependent protein kinase A (PKA) have been implicated in prostate cancer progression and AR signaling [11]. In particular, sympathetic stimulation of ADRB2 has been shown to potentially sensitize AR in cell lines under androgen depleted conditions [12], suggesting that ADRB2 might play a role in the development of CRPC. Furthermore, a number of target genes are common for the androgen and the PKA signaling cascades [13], and in steroidogenic cells both cAMP and PKA have been shown to regulate transcription of steroidogenic genes such as CYP17 and STAR [14-16], as well as to modulate their activity at the protein level [17].

While most pre-clinical evidence points towards a tumor promoting role of β-adrenergic signaling [18, 19], a previous study by Yu et al. reported an inverse correlation between ADRB2 expression levels and prostate cancer progression [20]. Low levels of ADRB2 in prostate cancer tissue were found to correlate with biochemical recurrence measured as increasing prostate-specific antigen (PSA) levels, or metastatic disease after radical prostatectomy. Conversely, our group has recently reported an association between the use of β-blockers (ADRB antagonists) and improved prostate cancer specific survival both for patients who have undergone ADT [21] and for patients with high risk or metastatic disease [22].

Our knowledge about the potential role of the ADRB2 in prostate cancer and CRPC development is still limited. Therefore, in this study, we have addressed this topic by performing immunohistochemical analyses and investigated the potential role of ADRB2 in development of CRPC in ADRB2 knockdown cell lines.

## RESULTS

### Low ADRB2 expression level in tumor tissue is associated with poor prognosis after androgen deprivation therapy

**Figure 1:**
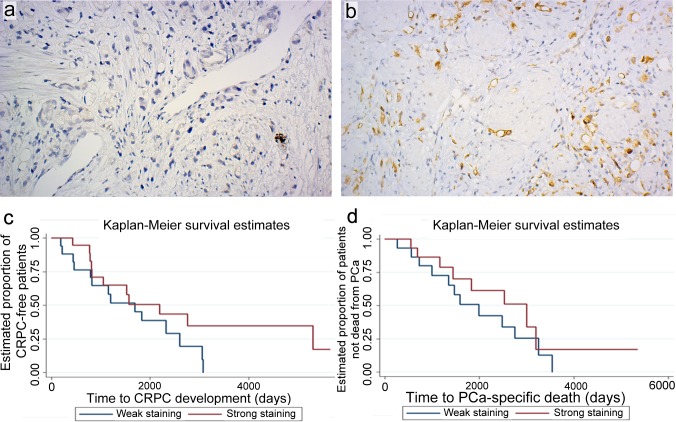
ADRB2 level is positively correlated with time to CRPC development Immunohistochemical analysis of ADRB2 expression in a TMA of transurethral resections of the prostate (TUR-P). Examples of tissue cores of Gleason score 9 tumors showing negative **a.** or strong staining **b.** intensity (original magnification 20x). Kaplan-Meier plots showing time to CRPC development **c.**, and time to prostate cancer (PCa)- specific death **d.** following TUR-P in patients stratified according to strong and weak staining intensity of ADRB2.

Tissue from 45 prostate cancer patients who had received hormonal therapy and had been treated with transurethral resection of the prostate (TUR-P) at Oslo University Hospital, Aker (the Oslo ADT cohort) were included in a tissue micro-array study. Five patients were excluded due to lack of cancerous tissue following staining with anti-ADRB2 antibody. The mean follow-up from initiation of ADT for the 40 patients included in the survival analyses was 71 months. For prostate cancer- specific mortality the mean follow-up was 70 months, as we lacked information on the cause of death for four patients. Patient and tumor characteristics at time of diagnosis are shown in Supplementary Table 2. Examples of negative and strong ADRB2 staining of two specimens with Gleason score 9 are shown in Figure 1a and 1b. Kaplan-Meier plots showing time to CRPC development and prostate cancer- specific mortality in patients stratified according to staining intensity above and below mean are shown in Figure 1c and 1d. Competing risk regression modelling showed that increasing staining intensity was associated with increased time to CRPC development, with an adjusted SHR of 0.67 (95% CI 0.46-0.97, *p*-value 0.035; adjusted for age at initiation of ADT and Gleason score) (Table 1). For prostate cancer- specific mortality, the association was not statistically significant (adjusted SHR 0.70, 95% CI 0.42-1.15, *p*-value 0.16). ADRB2 levels had no impact on all-cause mortality (adjusted HR 0.91, 95% CI 0.61-1.37, *p*-value 0.66).

**Table 1:** Uni- and multivariable HRs/SHRs for ADRB2 staining intensity and CRCP development and prostate cancerspecific and all-cause mortality

	Cumulative incidence	Increasing ADRB2 staining intensity	
		Crude estimate SHR/HR (95 % CI) *p*-value	Multivariable analysis^a^ SHR/HR (95% CI) *p*-value
Development of CRPC^b^	27/40	0.77 (0.53-1.13) 0.18	0.67 (0.46-0.97) 0.035
Prostate cancer- specific mortality^b^	21/35	0.71 (0.47-1.08) 0.11	0.70 (0.42-1.15) 0.16
Overall mortality	36/40	0.74 (0.53-1.04) 0.082	0.91 (0.61-1.37) 0.66

a Adjusted for age at initiation of androgen deprivation therapy and highest Gleason score from HE-slides of the TMA

b Analyzed by competing risk regression

A correlation analysis indicated no association between ADRB2 expression level and duration of ADT before TUR-P surgery (correlation coefficient-0.21, *p*-value 0.23).

### LNCaP shADRB2-tumors grow more rapidly in castrated mice

**Figure 2:**
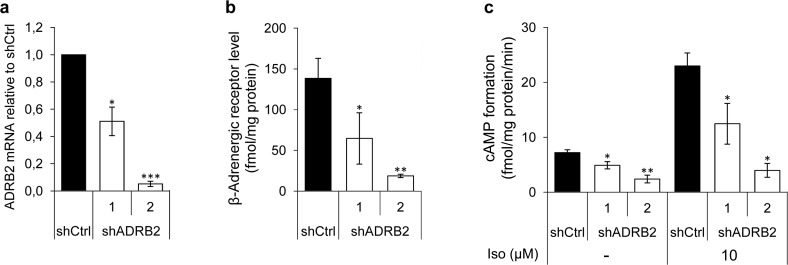
ADRB2 level, receptor binding, and downstream signaling activity in LNCaP shADRB2 cell lines **a.** ADRB2 mRNA levels were semi-quantitatively measured in RNA isolated from two LNCaP shADRB2 (shADRB2-1 and shADRB2-2) cell lines and a non-targeting shRNA LNCaP cell line (shCtrl) using Real-Time RT-PCR. Mean, ΔΔC_t_ calculated values relative to shCtrl cells are shown. **b.** β-adrenergic receptor level was quantified by determination of ^125^I-CYP specific binding to membrane protein fractions isolated from two LNCaP shADRB2 cell lines and shCtrl cells. Bars represent β-adrenergic receptor level reported as fmol/mg protein in the membrane fraction. **c.** Adenylyl cyclase activities in membranes isolated from LNCaP shADRB2 and shCtrl cells treated with vehicle or 10 μM isoproterenol were measured. The bars represent mean rate of formation of cAMP normalized to total protein in the membrane fractions (fmol/mg protein/min). All experiments were performed in biological triplicates (*n* = 3), mean ± standard deviation (SD). Statistical significance is indicated by asterisks (*: *p* < 0.05; **: *p* < 0.01; ***: *p* < 0.001).

Aiming to reveal potential mechanisms explaining the observed correlation between ADRB2 expression and time to CRPC development, we stably transfected LNCaP cells with shRNA plasmids targeting ADRB2 mRNA, yielding two knockdown cell lines (shADRB2-1 and 2), as well as a non-targeting shRNA plasmid (shCtrl). Real-Time RT-PCR analyses on mRNA isolated from shADRB2 and shCtrl cells revealed a 50% and 95% reduction of ADRB2 mRNA in shADRB2-1 and shADRB2-2, respectively, compared to shCtrl (Figure 2a). Radiolabeled ligand-binding assay measuring ^125^I-cyanopindolol (CYP)-binding to membrane-bound ADRBs confirmed the knockdown, with 50% and 85% lowered ADRB binding activity in shADRB2-1 and shADRB2-2 cells, respectively (Figure 2b). The receptor acts primarily through stimulating adenylyl cyclase (AC) activity, resulting in increased cAMP levels. The basal (non-stimulated) rate of conversion of [α-^32^P]ATP to [^32^P]cAMP was significantly lowered in both shADRB2-1 and 2 as shown in Figure 2c. Furthermore, stimulation with the non-selective ADRB-agonist isoproterenol showed a larger absolute and relative increase in adenylyl cyclase activity in shCtrl compared to both shADRB2 cell lines, indicating a functional effect of reduced ADRB2 levels.

**Figure 3:**
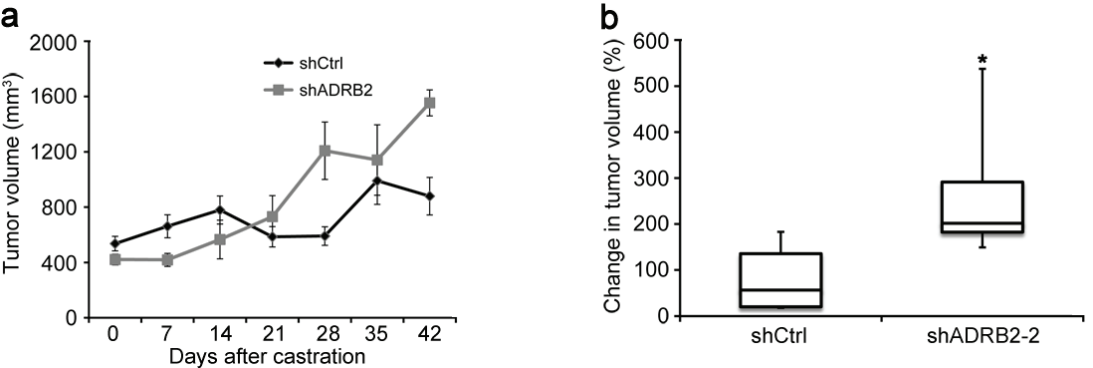
LNCaP shADRB2 xenograft tumors grow more rapidly than shCtrl tumors in castrated mice LNCaP shADRB2-2 and shCtrl cells were implanted subcutaneously into nude NOD-SCID mice. Once tumors reached 500 mm^3^ in size, mice were surgically castrated and taken off testosterone supplementation. Tumor volumes were measured weekly for 6 weeks. The graph **a.** shows mean (*n* = 10 for shADRB2-2 and 11 for shCtrl) tumor volumes (mm^3^) ± SEM. **b.** Box-and-whisker plot showing the percentage change in tumor volume 42 days after castration in NOD-SCID mice injected with LNCaP shADRB2-2 and shCtrl cells. Statistical significance was measured by Fischer exact test, and is indicated by asterisks (*: *p* < 0.05).

LNCaP shADRB2-2 and shCtrl cells were injected into NOD-SCID mice. The mice were castrated when the tumor diameter reached 10-12 mm and the tumor growth was followed in castrated mice for up to 42 days. After a brief lag period, the shADRB2-2 tumors grew more rapidly after castration, as shown in Figure 3a. Although the ten mice in the shADRB2-2 group had non-significantly smaller tumors than the eleven mice in the shCtrl group at time of castration, the shADRB2-2 tumors were larger 28 days after castration. The change in tumor volume from day 0 to day 42 was 3.5 fold higher in the shADRB2-2 compared to the shCtrl group (Figure 3b).

### Knockdown of ADRB2 in LNCaP cells is associated with reduced androgen glucuronidation activity

**Figure 4:**
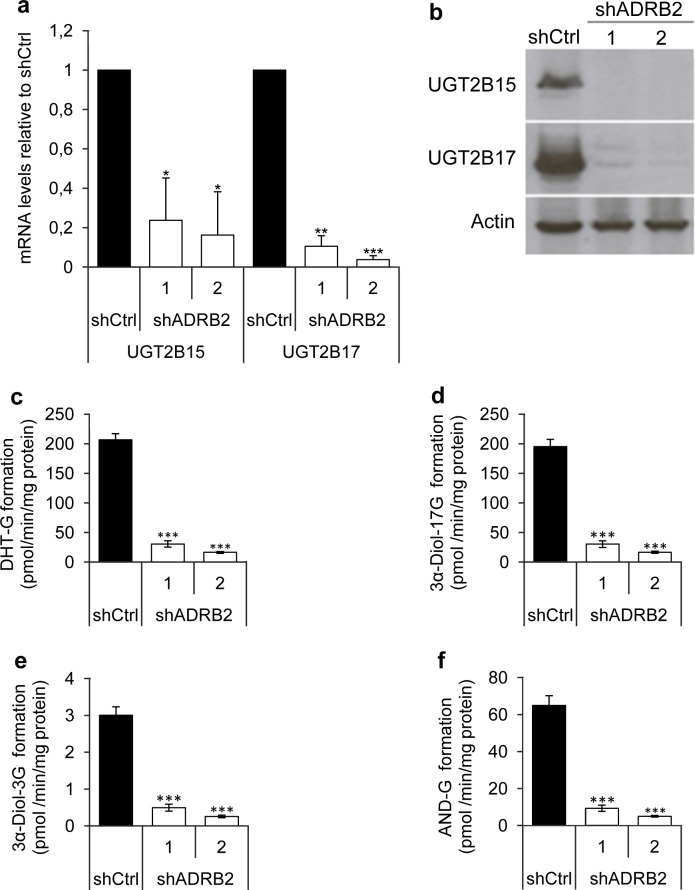
UGT2B15 and UGT2B17 mRNA, protein and effects on androgen glucuronide formation **a.** UGT2B15 and UGT2B17 mRNA levels were measured in RNA isolated from LNCaP shADRB2 (shADRB2-1 and shADRB2-2) and shCtrl cells using Real-Time RT-PCR. Bars represent mean, ΔΔC_t_ calculated values relative to shCtrl cells (*n* = 3) ± SD. **b.** UGT2B15 and UGT2B17 protein levels were visualized in cell homogenates by immunoblotting using anti-UGT2B15 and anti-UGT2B17 antibodies. Anti-actin antibodies were simultaneously used on the same homogenates to ensure similar loading on the lanes. **c.**-**f.** Cell homogenates from two LNCaP shADRB2 cell lines (shADRB2-1 and shADRB2-2) and shCtrl LNCaP cells (shCtrl) were mixed with uridine diphosphate glucuronic acid (UDPGA) and either dihydrotestosterone (DHT), 3α-androstanediol (3α-Diol) or androsterone (AND), for one hour, and levels of glucuronidated (G) androgens (c: DHT-G; d: 3α-Diol-17G; e: 3α-Diol-3G; f: AND-G) were measured by LC-MS/MS. The results are shown as mean formed glucuronide related to total protein in the homogenates (pmol/min/mg protein) from duplicated reactions on three biological replications ± SD. Statistical significance is indicated by asterisks (*: *p* < 0.05; **: *p* < 0.01; ***: *p* < 0.001).

We performed gene expression profiling of the LNCaP shADRB2 and shCtrl cells to aid in elucidating potential mechanisms explaining the association between ADRB2 and CRPC development, as well as the increased growth of the shADRB2 xenograft tumors. From this microarray analysis we observed differential expression of UDP-glucuronosyltransferase 2B15 and 2B17 in shADRB2 cells compared to the shCtrl cells (data not shown). To corroborate the microarray data, we performed Real-Time RT-PCR which showed that UGT2B15 was down-regulated 5-fold and 6-fold, and UGT2B17 down-regulated 10-fold and 20-fold, in shADRB2-1 and 2 respectively, relative to shCtrl (Figure 4a). The UGT2B15 and UGT2B17 protein levels were visualized by immunoblotting analysis. Whereas both proteins showed strong bands in shCtrl cells, UGT2B15 and UGT2B17 were virtually un-detectable in both shADRB2 cell lines (Figure 4b).

Furthermore, lowered UGT2B15 and UGT2B17 expression was accompanied by reduced androgen glucuronide formation (Figure 4c-4f). Dihydrotestosterone-glucuronide (DHT-G), two androstanediol glucuronides (3α-Diol-17G, 3α-Diol-3G) and androsterone glucuronide (AND-G) formation was strongly reduced in the shADRB2 cell lines compared to shCtrl cells, with a steady 85% lowering of glucuronide formation in shADRB2-1 cells, and a 95% fold lowering in shADRB2-2 cells. Glucuronidation activity in positive (human liver homogenates) and negative (HEK293 cell homogenates) controls is shown in Supplementary Figure 1.

**Figure 5:**
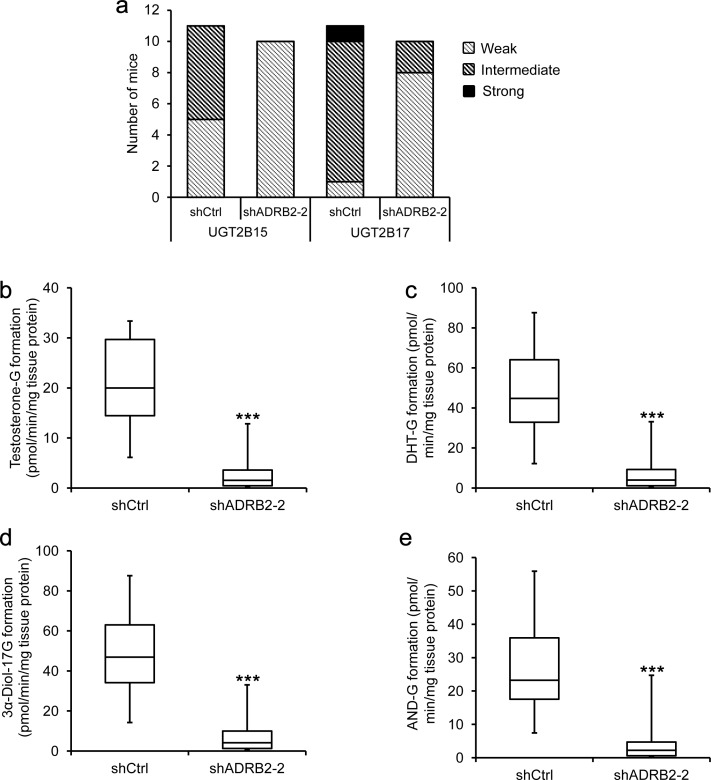
LNCaP shADRB2 castrated mouse tumor characteristics and glucuronidation activity **a.** Excised xenograft tumors were formalin-fixed and paraffin-embedded, and sections were stained with anti-UGT2B15 (1:500) and anti-UGT2B17 (1:500) antibodies. Frequencies of staining intensities (weak, intermediate and strong) from tumors derived from mice injected with shCtrl (*n* = 11) and shADRB2-2 (*n* = 10) LNCaP cells are shown. **b.**-**e.** Fresh frozen tumor tissue from the same mice were homogenized and added UDPGA and either dihydrotestosterone (DHT), 3α-androstanediol (3α-Diol) or androsterone (AND) in a glucuronidation assay. Formation of steroid glucuronides (c: DHT-G; d: 3α-Diol-17G; e: 3α-Diol-3G; f: AND-G) was measured by LC-MS/MS. The results are shown as a box-and-whisker plot showing formed glucuronide related to total protein in the tissue homogenates (pmol/min/mg tissue protein) from triplicate reactions on the same homogenates. Statistical significance is indicated by asterisks (**: *p* < 0.01; ***: *p* < 0.001).

These findings led us to investigate whether castration of mice injected with LNCaP shCtrl or shADRB2-2 cells had an effect on the expression and activity of UGT2B15 and UGT2B17 *in vivo*. Immunohistochemical staining of tumor tissue from the xenograft study using anti-UGT2B15 and anti-UGT2B17 antibodies showed that the phenotypic differences between shCtrl and shADRB2-2 cells were maintained also after castration (Figure 5a). UGT2B15 and UGT2B17 staining intensities were statistically significantly higher in shCtrl tumors than shADRB2-2 tumors (*p* = 0.006 and *p* = 0.0004 for UGT2B15 and UGT2B17, respectively). UGT2B17 negatively correlated to average daily growth of the tumors (correlation coefficient -0,518, *p* = 0.016), whereas UGT2B15 did not (correlation coefficient -0.188, *p* = 0.41). Furthermore, the glucuronidation activity in tumor extracts was on average 85% lower in shADRB2 xenograft mice compared to shCtrl mice (Figure 5b-5e).

### Knockdown of ADRB2 improves androgen responsiveness *in vitro*

**Figure 6:**
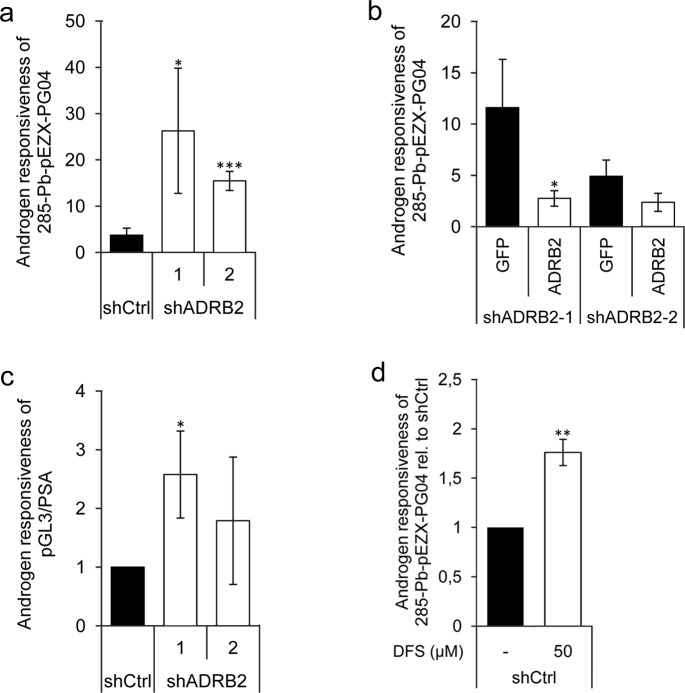
Increased androgen responsiveness in LNCaP shADRB2 cell lines **a.** LNCaP shADRB2-1, shADRB2-2 and shCtrl cells were transfected with the androgen responsive element-containing luciferase reporter construct 285-Pb-pEZX-PG04. The following day, cells were incubated in hormone-deprived medium containing 2% CSS supplemented with either 10 nM DHT or vehicle and further incubated for 48 hours. The androgen responsiveness of 285-Pb-pEZX-PG04 in LNCaP shADRB2-1, shADRB2-2, and shCtrl cells are shown relative to vehicle treated (n = 3) ± SD. **b.** LNCaP shADRB2-1 and shADRB2-2 were transfected with the reporter construct 285-Pb-pEZX-PG04 and either an ADRB2 expression vector (pCDNA3.1-ADRB2) or a control expression vector (pEGFP-C3). Mean androgen responsiveness relative to vehicle treated cells is shown (n = 3) ± SD. **c.** Cells were transfected with a reporter plasmid including the 5′-regulatory region of PSA (pGL3/PSA), and the cells were stimulated as described in (a). Androgen responsiveness is given as the relative luciferase activities from DHT-stimulated cells normalized to vehicle-treated cells from three independent experiments (*n* = 3) mean ± SD. **d.** shCtrl cells were transfected with 285-Pb-pEZX -PG04 and were either treated with 50 μM diclofenac or with vehicle and then half of the cells were stimulated with 10 nM DHT the following day and all cells were harvested after 72 hours. Mean DHT responses from three independent experiments are shown relative to un-stimulated shCtrl cells (given value 1.0) ± SD treated with either diclofenac or vehicle ± SD. Statistical significance is indicated by asterisks (*: *p* < 0.05; **: *p* < 0.01; ***: *p* < 0.001).

After confirming that lowered ADRB2 expression lead to a change in glucuronidation activity, we were interested in finding out whether this could provoke a change in the AR activity in the cells. DHT-stimulated LNCaP shADRB2 and shCtrl cells transiently transfected with the probasin-based promoter and luciferase reporter construct 285-Pb-pEZX-PG04 revealed that shADRB2 cells had a higher relative androgen responsiveness than shCtrl cells (7-fold and 4-fold in shADRB2-1 and 2, respectively) (Figure 6a). Similar results were obtained when the cells were pre-incubated for 96 hours in hormone-deprived medium prior to stimulation (data not shown). To test if the effect was caused by reduced level of ADRB2, we rescued ADRB2 expression in shADRB2-1 and 2 using pCDNA3.1-ADRB2, constitutively expressing the ADRB2 gene. The relative luciferase activity was decreased by 80% and 50%, yielding the knockdown cells more similar to shCtrl cells (Figure 6b). Furthermore, we wanted to test if the effect observed with a probasin-based promoter (285-Pb-pEZX-PG04) could be replicated with a different androgen responsive reporter plasmid containing 7 kb of the 5′ upstream region of the PSA promoter (pGL3/PSA). We transfected shADRB2 and shCtrl cells with pGL3/PSA and measured relative androgen responsiveness after stimulation with 10 nM DHT or vehicle for 48 hours. As with 285-Pb-pEZX-PG04, the androgen response of pGL3/PSA was higher when transfected into shADRB2 cells than shCtrl cells (2.5- and 1.8-fold higher in shADRB2-1 and shADRB2-2, respectively) (Figure 6c).

Next, we were interested in seeing whether the androgen responsiveness could be modulated by inhibiting UGT2B15 and UGT2B17 activity. We treated LNCaP shCtrl cells with the UGT2B substrate diclofenac sodium (DFS), which competitively inhibits UGT2B-action towards androgens. Stimulation with 50 μM diclofenac sodium induced a statistically significant 1.8-fold rise in normalized 285-Pb-pEZX-PG04-driven luciferase activity (Figure 6d).

The androgen responsiveness was non-significantly higher in the shADRB2-1 cell line than in the shADRB2-2 cell line (Figure 6a). This might be due to the fact that the androgen receptor is slightly induced in the shADRB2-1 cell line (measured by western immunoblotting of protein extracts; Supplementary Figure 2). The androgen receptor was not up-regulated in shADRB2-2 cells compared to shCtrl.

### Prostate-specific antigen responsiveness is increased in ADRB2 knockdown LNCaP cells

**Figure 7:**
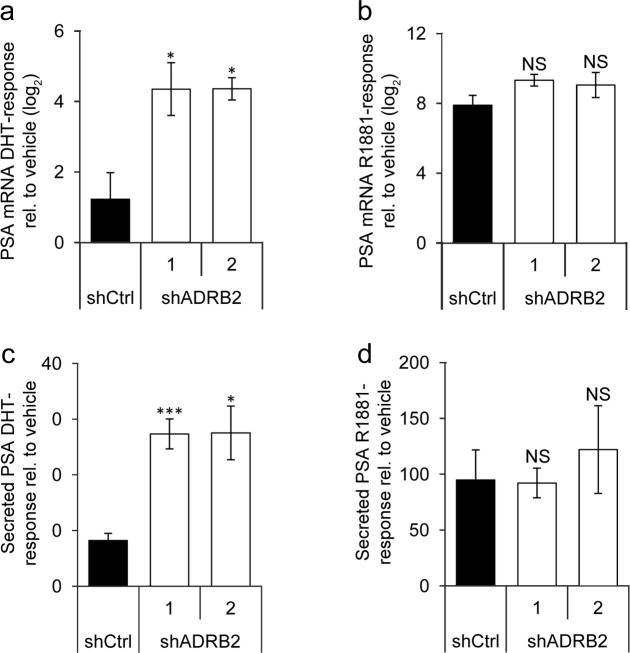
Prostate-specific antigen responsiveness is higher in shADRB2 than in shCtrl cells **a.**, **b.** LNCaP shADRB2 and shCtrl cells were starved in 2% CSS for 96 hours prior to stimulation with 1 nM DHT or 1 nM R1881 for 48 hours. RNA was harvested and analyzed for PSA/KLK3 mRNA expression by Real-Time RT-PCR. Gene expression upon stimulation with **a.** DHT and **b.** R1881 relative to non-stimulated cells (vehicle) was calculated by the ΔΔC_t_- method. Bars represent log_2_-transformed androgen responses (*n* = 3) ± SEM. **c.**, **d.** Secreted total PSA (TPSA) was measured in medium samples from cells stimulated with **c.** DHT and **d.** R1881 by time-resolved fluorescence, and was related to non-stimulated cells (vehicle). Bars represent androgen responses (*n* = 4) mean ± SD. NS: non-significant difference from shCtrl.

We hypothesized that increased reporter-driven androgen responsiveness would be mirrored by an increase in the PSA response upon androgen stimulation. shADRB2 and shCtrl cells were pre-incubated in hormone-deprived medium for 96 hours, and then stimulated for 48 hours with either 1 nM DHT, 1 nM R1881, or vehicle, before harvesting and isolating total RNA. The Real-Time RT-PCR reaction revealed that DHT induced a significantly more pronounced response on PSA mRNA in both shADRB2 cell lines than in shCtrl (Figure 7a). Stimulation with the non-glucuronidable synthetic androgen R1881 resulted in a greater response in all the cells compared to DHT, but there were no significant differences between shADRB2 and shCtrl cells (Figure 7b). To substantiate these findings, we performed a similar experiment where we measured secreted PSA in medium from androgen-stimulated cells. Figure 7c shows the androgen responsiveness from cells stimulated with DHT, and Figure 7d shows the relative responses acquired with R1881. As with PSA mRNA, only stimulation with DHT yielded a significant difference in increased PSA-response between shADRB2 cell lines and the shCtrl cell line.

### Glucuronidation activity affects androgen levels in LNCaP ADRB2 knockdown cells

**Figure 8:**
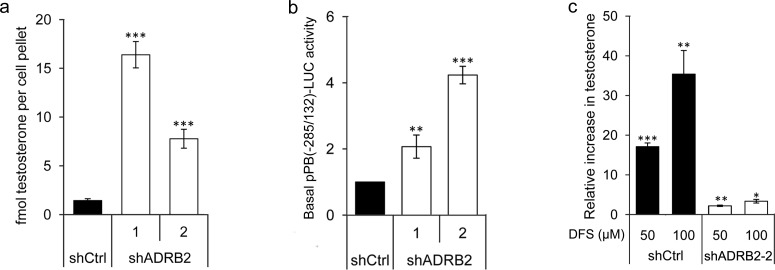
Reduced androgen glucuronidation activity affects the level of bioactive androgen *in vitro* **a.** The basal level of testosterone was measured in shCtrl, shADRB2-1, and shADRB2-2 LNCaP cells cultured in FCS medium. Steroids were extracted from the cells, dried, reconstituted, and run on an LC-MS. Integrated, internal standard (IS)-normalized mean peak areas are shown related to equal cell pellets. **b.** LNCaP shADRB2-1, shADRB2-2 and shCtrl cells were transfected with pPB(−285/132)-LUC. After 72 hours incubation in FCS-medium, basal luciferase activity of the pPB(−285/132)-LUC reporter was measured and related to SEAP. The results are shown as mean, basal luciferase activities related to shCtrl (given value 1.0) ± SD from three independent experiments. **c.** LNCaP shADRB2-2 and shCtrl cells were treated with 50 or 100 μM diclofenac or vehicle for 48 hours. Integrated, internal standard (IS)-normalized peak areas were related to total protein content and is presented as mean relative increase compared to vehicle treated cells (given value 1.0) (nM testosterone/mg protein) from three independent experiments ± SD. Statistical significance is indicated by asterisks (*: *p* < 0.05, **: *p* < 0.01, ***: *p* < 0.001).

A plausible effect of lowered glucuronidation activity is shifting of the substrate/glucuronide homeostasis and subsequent accumulation of glucuronidable androgens, which could help explain the observed increase in androgen responsiveness in shADRB2 cells. We therefore measured intracellular testosterone levels in shCtrl and shADRB2 cells cultured in FCS medium for 48 hours (Figure 8a). An 11-fold and 5.5-fold higher testosterone level was found in shADRB2-1 and shADRB2-2, respectively, compared to shCtrl. The basal reporter activity driven by the androgen responsive probasin promoter (pPB(−285/132)-LUC) was higher in shADRB2 cell lines compared shCtrl cells (Figure 8b) supporting that the testosterone level is increased in shADRB2 cell lines.

Furthermore, to establish a link between glucuronidation activity and levels of bioavailable androgen, we supplemented shCtrl and shADRB2-2 cells with diclofenac sodium and measured the intracellular testosterone levels. Diclofenac sodium caused a dose-dependent induction in testosterone levels, with 17-fold and 35-fold induction with 50 and 100 μM DFS, respectively (Figure 8c). Comparably, diclofenac had only a minor effect in shADRB2-2, which has 95% lower androgen glucuronidation activity than shCtrl cells.

### UGT2B15 and UGT2B17 are correlated to ADRB2 in two patient material data sets

Initially, as we observed a regulation of UGT2B15 and UGT2B17 levels after ADRB2 knockdown *in vitro*, we wanted to examine if these proteins were correlated with ADRB2 expression in the Oslo ADT cohort. Both UGT2B15 and UGT2B17 predominantly showed cytoplasmic staining of luminal cells (Supplementary Figure 3). As can be seen in Table 2, UGT2B15 staining was found to be positively correlated with ADRB2 staining (correlation coefficient 0.39, *p*-value 0.001). A similar trend was found for UGT2B17; however this was not statistically significant.

**Table 2:** Spearman's rank correlations between ADRB2 and UGT2B15 and UGT2B17

		ADRB2 versus UGT2B15		ADRB2 versus UGT2B17	
TMA study	Cohort	No. of pairs	Correlation (95% CI) *p*-value	No. of pairs	Correlation (95% CI) *p*-value
Oslo ADT		65	0.39 (0.16-0.59) 0.001	64	0.19 (−0.066-0.42) 0.13
Vancouver Prostate Centre Tissue Bank	All cancer cases	583	0.40 (0.33-0.47) <0.0001	602	0.35 (0.27-0.42) <0.0001
	Recurrent PCa	209	0.50 (0.38-0.59) <0.0001	214	0.33 (0.20-0.45) <0.0001
	CRPC	58	0.64 (0.45-0.78) <0.0001	58	0.33 (0.074-0.55) 0.011

To verify the results in an independent study, immunohistochemical stainings with ADRB2, UGT2B15 and UGT2B17 were performed on four TMAs, merged into a single data set, from the Vancouver Prostate Centre Tissue Bank. Of a total of 306 patients, 262 had cores positive for cancer. Among these, 96 patients had recurrent prostate cancer, 23 had diagnosed CRPC, and the remaining 143 were patients who have undergone radical prostatectomy. In this cohort, we observed a correlation between ADRB2 and UGT2B15 similar to the correlation in the Oslo ADT cohort (Table 2). Interestingly, the correlation was stronger when only samples from patients who have experienced recurrence or who have developed castration resistant prostate cancer were included in the analysis. In this dataset, the UGT2B17 staining was also significantly correlated with ADRB2 staining (Table 2).

Finally, we performed a competing risk regression analysis to see if UGT2B15 and UGT2B17, like ADRB2, were associated with development of CRPC in the Oslo ADT cohort. 33 and 34 out of the 45 patients had successful immunohistochemical staining of tumor tissue with UGT2B15 and UGT2B17, respectively. Weak UGT2B15 staining intensity was significantly associated with a more rapid development of CRPC (SHR 0.39, 95% CI 0.16-0.97, *p*-value 0.043), while UGT2B17 was not (Table 3). Neither UGT2B15 nor UGT2B17 were associated with prostate cancer specific or all-cause mortality (Table 3a and 3b, respectively).

**Table 3:** A) Uni- and multivariable HRs/SHRs for UGT2B15 staining intensity and CRCP development and prostate cancer- specific and all-cause mortality. B) Uni- and multivariable HRs/SHRs for UGT2B17 staining intensity and CRCP development and prostate cancer- specific and all-cause mortality

**A)**	**Cumulative incidence**	**Increasing UGT2B15 staining intensity**	
		**Crude estimate SHR/HR (95 % CI) *p*-value**	**Multivariable analysisa SHR/HR (95% CI) *p*-value**
Development of CRPC^b^	22/33	0.63 (0.32-1.25) 0.19	0.39 (0.16-0.97) 0.043
Prostate cancer- specific mortality^b^	15/28	0.63 (0.30-1.32) 0.22	0.38 (0.09-1.59) 0.19
Overall mortality	29/33	0.67 (0.37-1.22) 0.19	0.90 (0.42-1.95) 0.80
**B)**	**Cumulative incidence**	**Increasing UGT2B17 staining intensity**	
		**Crude estimate** **SHR/HR (95 % CI) *p*-value**	**Multivariable analysisa** **SHR/HR (95% CI) *p*-value**
Development of CRPC^b^	23/34	1.06 (0.55-2.05) 0.87	0.87 (0.43-1.73) 0.69
Prostate cancer- specific mortality^b^	16/29	0.89 (0.46-1.71) 0.72	0.69 (0.41-1.16) 0.16
Overall mortality	30/34	0.93 (0.52-1.67) 0.80	1.17 (0.60-2.30) 0.65

a Adjusted for age at initiation of androgen deprivation therapy and highest Gleason score from HE-slides of the TMA

b Analyzed by competing risk regression

## DISCUSSION

We report that prostate cancer patients expressing low levels of the β_2_-adrenergic receptor in the cancer tissue more rapidly develop castration-resistant prostate cancer. Furthermore, xenograft tumors from prostate cancer cells with knockdown of ADRB2 were shown to grow more rapidly in castrated mice than xenografts tumors from control cells. We have identified a novel mechanism by which ADRB2 may indirectly regulate the activity of the androgen receptor in prostate cancer cells, namely through regulating glucuronidation, a critical step in the extrahepatic phase II metabolic elimination pathway of androgens. LNCaP cells expressing low ADRB2 levels showed reduced UGT2B15 and UGT2B17 expression and activity compared to cells expressing high ADRB2 levels. The low ADRB2 expressing LNCaP cells were more responsive to androgen stimuli, displayed increased testosterone levels and a higher basal androgen receptor activity. Furthermore, supplementation with the competitive UGT2B-substrate diclofenac enhanced androgen responsiveness in high-ADRB2 expressing LNCaP cells, with a simultaneous increase in the intra-cellular testosterone level.

Like androgens, adrenergic stimulation contributes to prostatic differentiation *in vivo* [23]. Moreover, ADRB2 signaling activates androgen responsive promoters *in vitro* and is therefore suggested to play a role in development of CRPC [12]. Short-term activation of ADRB2 stimulates androgen receptor activity [12], while long-term activation of ADRB2 leads to desensitization of ADRB2 [24]. Furthermore, down-regulation of ADRB2 induces de-differentiation and epithelial to mesenchymal transition (EMT) [20], a process associated with CRPC development. This is the first study addressing whether ADRB2 correlates with CRPC *in vivo,* and the data are consistent with the hypothesis that ADRB2 is associated with CRPC.

This study points to a novel mechanism by which long-term knockdown of ADRB2 may support CRPC development. In xenografts, LNCaP tumors expressing low levels of ADRB2 have a shorter lag period and grow more rapidly after castration than tumors with normal ADRB2-levels, indicating that these cells may be more adapted to an androgen-deprived milieu. Being more adapted to castration theoretically predicts therapy failure or imminent recurring growth, which seems to be the case for these shADRB2 tumors.

The increased testosterone levels and enhanced androgen responsiveness observed in ADRB2 knockdown cells may relate to the observation that androgen-glucuronidating activity is down-regulated. Reducing glucuronidation could preserve residual and *de novo* biosynthesized androgens and thus rescue androgen receptor stimulation, which would give the cells an “edge” in an androgen-deprived micro milieu. Thus, this may represent an adaption mechanism by which the cells maintain a sufficient androgen receptor activity to uphold survival. *In vivo,* this mechanism may complement the well-known increase in intra-tumoral androgen biosynthesis and androgen receptor expression observed in CRPC [25-27].

The cAMP signaling pathway is an essential inducer of steroidogenesis in steroidogenic cells [14-17]. To what extent β-adrenergic signaling regulates steroid synthesis in prostate cancer cells is not known, but our study suggests that the receptor may be involved in regulating the amount of bioactive androgen through modulating glucuronidation activity. Testosterone levels were increased both in cells expressing low levels of UGT2B15 and UGT2B17, and in cells treated with diclofenac, which has previously been reported to be a UGT2B15 and UGT2B17 competitive inhibitor [28-30]. Furthermore, stimulation with the synthetic androgen R1881, reported to be non-glucuronidable [31], gave similar androgen responses in the shADRB2 (low UGT2B) and shCtrl (high UGT2B) cell lines, indicating that glucuronidation regulates the observed differences in androgen responsiveness solitarily. In support of this, a study by Chouinard et al., showed that knockdown of UGT2B15 and UGT2B17 in LNCaP cells lead to a more pronounced modulation of androgen-regulated genes [32].

Several studies have investigated the expression level of UGT2B15 and UGT2B17 in hormone naïve and castration resistant prostate cancer [9, 25, 33]. Collectively, none of these studies show a significant difference in immunohistochemical staining intensity for neither UGT2B15 nor UGT2B17 expression between androgen-dependent prostate cancer and CRPC. No study has yet, however, investigated how UGT2B15- or UGT2B17 expression in hormone naïve prostate cancer relates to time to development of CRPC. In our analyses, UGT2B15 staining intensity was statistically significantly correlated with CRPC development, while UGT2B17 was not. Both enzymes, however, are positively correlated with ADRB2 staining, which itself was associated with CRPC development. The positive correlations between the two UGT2Bs and ADRB2 in tissue samples support our observations of reduced UGT2B15 and UGT2B17 levels after knockdown of ADRB2 in LNCaP cells. Furthermore, the finding that differences in glucuronidation activity between shCtrl and shADRB2 cells were maintained in our mouse model after castration, points to the possibility that ADRB2 may influence glucuronidation activity also in humans.

If UGT2B15 and UGT2B17 are important determinants of the availability of bioactive androgens in the tumor micro milieu *in vivo*, patients with low ADRB2 expression may have a lower response to androgen-deprivation therapy through lowered UGT2B15 and UGT2B17 protein levels. This might explain our observation that low ADRB2 levels are associated with a poor prognosis.

It should be noted that diclofenac is a non-steroidal anti-inflammatory drug, and may thus affect pathways that directly or indirectly affect androgen receptor activity, i.e. through affecting prostaglandin metabolites that are known to inhibit AR [34]. Whether this in turn could affect our reported effects on androgen responsiveness was not assessed in this study.

Low level of ADRB2 has previously been shown to predict a shorter time to clinical failure after radical prostatectomy, as defined by biochemical recurrence [20]. It is worth noting, however, that 60-70% of men experience recurrence without emergence of clinical symptoms, and only around 8% of patients that experience biochemical recurrence die from prostate cancer [35]. Thus, clinical progression probably serves as a better end point in biomarker studies. We used clinical progression as an end point, and see that ADRB2 may act as a prognostic biomarker for CRPC. Furthermore, alterations in androgen glucuronidation activity are presented as one potential mechanism by which ADRB2 may regulate development of castration resistant prostate cancer.

## MATERIALS AND METHODS

### Ethics

The Regional Ethical Committee (s-04153c), the Data Protection Official at Oslo University Hospital (41-2009 AUS) and The Norwegian Data Protection Authority (09/00450-2 /bso) has approved this study. Written consent was obtained from all surviving patients, and a permission to include clinical information on deceased patients was obtained from the Regional Committee for Medical and Health Research Ethics (2009/1028).

### Patient material and TMA construction

For the Oslo ADT TMA, 61 patients treated with palliative transurethral resection of the prostate (TUR-P) and ADT at Oslo University Hospital, Aker, in the period 1992- 2008 were identified retrospectively from medical records. 16 patients were excluded from the analyses due to lack of sufficient tissue or lack of consent.

Clinical information was obtained from medical records. Retracted data included date of birth, date of diagnosis, date of initiation of hormonal treatment or orchiectomy, and date of progression. Date and cause of death were obtained from Statistics Norway, per May 1st 2012.

Tissue was obtained from “The Prostate Biobank- a resource for urological research in Norway” (No.119 The Biobank Registry at Norwegian Institute of Public Health). One area representing normal and two areas representing prostate cancer tissue were marked on hematoxylin/eosin stained sections, extracted using a 0.6 mm tissue core, and mounted using a semi-motorized tissue arrayer (TMABooster, Alphelys, Plaisir, France).

The treatment initiation date was set at the first time of administration of anti-androgen, luteinizing hormone- releasing hormone (LHRH) agonist, or date of orchiectomy, where applicable. Where the exact date of diagnosis, initiation of ADT or disease progression was not noted in the patient's journal, the actuar-method was used to assign an event date; that is, the middle date between two known dates before and after diagnosis, start of hormonal treatment, or disease progression.

Patients were considered to have CRPC in the case of two consecutive PSA rises, progression to metastatic disease, or when noted explicitly in the patients journal.

Briefly, for the TMA constructed from specimens obtained from the “Vancouver Prostate Centre Tissue Bank” [36], H&E-stained slides were inspected and desired areas of 1 mm were extracted and mounted manually (Beecher Instruments, MD, USA) as duplicated cores. Among the 304 prostate cancer specimens, 143 were from radical prostatectomies, 96 were from radically operated patients who had been pretreated with androgen deprivation therapy for one to twelve months prior to surgery, and the remaining 23 were CRPC samples obtained through TUR-P. The patients were operated in the period 1999-2009.

### Immunohistochemistry

For the Oslo ADT TMA, TMA sections of 4 μm were deparaffinized and antigens were retrieved at 97°C for 20 minutes using the PT-link (Dako, Glostrup, Denmark) and “Target Retrieval Solution, high pH” (K8004, Dako) for ADRB2 whereas the slide was microwaved for 10 min in citrate buffer (pH 6.0) (Thermo Fischer Scientific, Waltham, MA) and immersed in 0.5% v/v hydrogen peroxide/methanol for 20 min for UGT2B15 and UGT2B17 immunostaining. The anti-ADRB2 antibody (MC2656, MBL International, Woburn, MA), the anti-UGT2B15 antibody [37], and the anti-UGT2B17 antibody [38] were used in dilution 1:400, 1:500 and 1:500, respectively. The immunostainings were visualized using the Envision Flex” (K8010, Dako) kit. Images were captured using a Zeiss AXI0 Imager.A1 microscope with an attached Zeiss AxioCamERc5s camera (Zeiss, Oberkochen, Germany) using Histolab 8 (Alphelys, Plaisir, France), and manual scoring of the staining was performed by pathologists AS and WW (ADRB2) or AS and BK (UGT2B15 and UGT2B17). For survival analyses, staining intensity for the spot(s) with the highest apparent Gleason score was chosen for further analysis. Where the tissue in a spot showed more than one staining intensity, fractions were used. In the case of two spots with the same apparent Gleason score from one patient, or disagreement regarding staining intensity, the average intensity was calculated and used in the analyses. The Gleason scores were determined by two experienced uro-pathologists (AS and WW).

ADRB2 antibody specificity and sensitivity was tested, and is shown in Supplementary Material and Methods.

The immunohistochemical staining of TMAs in the “Vancouver Prostate Centre Tissue Bank” was performed as previously described [36]. Pathologist LF evaluated staining intensities, and the staining intensities in the dataset were exclusively used for correlation analyses.

For the immunohistochemical analyses of xenograft tumors, the paraffin-embedded tumors were in brief sectioned, mounted onto glass slides, and stained with UGT2B15 (1:500) or UGT2B17 (1:500) antibodies. Pathologist BK scored staining intensities.

### Plasmids

Two short hairpin SureSilencing^TM^ shRNA plasmids with insert sequences targeting ADRB2 mRNA, as well as a non-targeting shRNA as control, were purchased from Qiagen (Supplementary Table 1) (Qiagen, Hilden, Germany). Two androgen-responsive reporter constructs were used including the probasin and PSA promoters, respectively. The PSA reporter plasmid (pGL3/PSA) included 7 kb of the 5′- upstream region of PSA [39]. The probasin promoter sequence (pPB(−285/132)-LUC [40]) was cloned into a Gluc-ON^TM^ Promoter clone system (pEZX-PG04, GeneCopoeia) expressing Luc and Secreted alkaline phosphatase (SEAP) as tracking gene (pEZX-PG04-Pb-LUC). An empty pEZX-PG04 vector expressing SEAP was used to control for differences in transfection efficiency. A pCDNA3.1 vector, expressing the ADRB2 gene (pCDNA3.1-ADRB2), was used for over-expression [41].

### Cell lines

LNCaP cells (ATCC (VA, USA), purchased 10/2009), were maintained in RPMI 1640 containing 10% Fetal Bovine Serum (FBS) ((Sigma-Aldrich, St. Louis, MO), 100units/ml penicillin and 50mg/ml streptomycin (InVitrogen, Carlsbad, CA) at 37°C with 5% CO_2_ and humidified air, and were given fresh medium every 48 hours. Stable ADRB2 knockdown was achieved by transfection of LNCaP cells at passage 25 with three different shRNA sequences (two different ADRB2 shRNAs; shADRB2-1 and 2, and a non-targeting shRNA; shCtrl), using Dharmafect Duo (Dharmacon/GE). Stably transfected cells were maintained in 200μg/ml G418 sulphate. Cells were exclusively used between passage 28 and 45. Cell IDs of parental LNCaP, LNCaP shADRB2 and shCtrl cell lines were verified using the STR PowerPlex16 System (Promega, Fitchburg, WI)(tested 07/2014).

### Stimulation of cell cultures

To study effects of androgen stimulation, LNCaP cells were grown in phenol red free RMPI 1640 (Life technologies, Carlsbad, CA) added 2% charcoal-stripped FBS (CSS, Gibco, Carlsbad, CA) and supplemented with either 1.0 or 10 nM metribolone (R1881, Roussel UCLAF), 1.0 or 10nM dihydrotestosterone (DHT, kindly provided by the Hormone Laboratory, Oslo University Hospital), 50 μM diclofenac sodium (DFS, Cayman Chemical Company, MI) or vehicle (ethanol). The cells were preincubated in RPMI with 2% CSS where noted.

### Animal experiments

Twenty-one in-house bred, 4 week old male NOD-SCID gamma/null mice weighing 28.2 ± 4 g were administered 0.03mg/ml testosterone (Sigma-Aldrich) in drinking water one week prior to s.c. injection into the hind flank with 2 × 10^6^ LNCaP shADRB2-2 (*n* = 10) or shCtrl (*n* = 11) cells suspended in Matrigel (1:1) (BD Biosciences, San Jose, CA). Tumor volumes were assessed weekly using caliper measurements and calculated by the formula: (length × width^2^)/2. Once tumor size reached 500mm^3^, the mice were surgically castrated under anesthesia by removal of testes and taken off testosterone supplementation. The mice were sacrificed when the tumor volumes reached 2000 mm^3^. The tumors were excised and split in two parts: One part was fresh frozen and used for measurement of glucuronidation activities, the other formalin-fixed and paraffin-embedded for immunohistochemical analyses. The experiment was approved by the National Animal Research Authority (FOTS ref. 7132) and was performed according to regulations of the Federation of European Laboratory Animals Science Association.

### Transient transfection and reporter assays

The LNCaP sub-cell lines were transfected using the Dharmafect Duo reagent. Reporter activities (secreted luciferase and SEAP) in medium samples were measured 48 hours after transfection using the Secret-Pair Dual Luminescence Assay kit (GeneCopoeia) on a Victor Wallac Spectrophotometer (PerkinElmer, Waltham, MA). To determine intra-cellular luciferase activity cells, cells were lysed in 1X Reporter Lysis buffer (Promega), the supernatant mixed with Luciferase Assay Reagent (Promega) and the activity measured on a TD-20/20 luminometer (Turner Designs, Sunnyvale, CA).

### RNA extraction and real-time RT-PCR

Total RNA was extracted using the TRIzol reagent following manufacturers protocol (Invitrogen). 100 ng of total RNA was used in the qScript™ One-Step qRT-PCR Kit (Quanta Biosciences, Gaithersburg, MD). The RT-PCR reactions were performed on a CFX Connect™ Real-Time System (BioRad, Hercules, CA) under 48° C for 10 min; 95°C for 5 min for the first cycle; 95°C for 15 s, 55°C for 30 s for 40 cycles; 60 melt curve read offs from 65-95°C. ALAS-1 or POLR-2A mRNA expression were used for reference. To display relative gene expression, the ΔΔC_t_ formula [42] was used. The primers used were: ADRB2 fwd: gtcttgagggctttgtgctc, rev: ggcagctccagaagattgac; UGT2B15 fwd: gatcatcgaccccagagaaa, rev: tcactgtaaaccagccaaacc; UGT2B17 fwd: gatcatcgaccccagagaaa, rev: cgcccattcttaccaaatgt; Kallikrein 3/Prostate Specific Antigen (KLK3/PSA) fwd: ccctgagcacccctatcaac, rev: tgagtgtcggtgggttgtg.

### Prostate specific antigen (PSA/KLK3)

Total PSA in medium was determined by the AutoDELFIA ProStatus PSA Free/Total Kit (PerkinElmer Inc., USA) by time-resolved fluorescence on the AutoDELFIA instrument. Total PSA was normalized to the amount of protein in each sample.

### Protein extraction and immunoblot analysis

The cells were harvested, lysed in whole cell buffer [43], and centrifuged at 16,000 g for 20 min. Immuno-blots were prepared and visualized as previously reported [37, 38]. Anti-UGT2B15 (1:1500) or anti-UGT2B17 (1:2000) was used as primary antibodies, and anti-actin (1:2000, #A5060, Sigma-Aldrich) as loading control. Both UGT2B15 and UGT2B17 antibodies were kindly provided by A. Bélanger (CHU-Québec research centre) [38].

### Radioligand binding and adenylyl cyclase assays

Cell membrane fractions were prepared as described in [44], and the ADRB2 protein binding activity was measured by radioligand binding assay, as previously described [45] with a binding buffer described in [46]. The total number of specific binding sites was determined. Ligand binding was normalized to the total amount of protein in the membrane fractions.

Adenylyl cyclase activity was measured by determining conversion of [α-^32^P]ATP to [^32^P]cAMP in cell membrane fractions in the presence and absence of 10 μM isoproterenol (Sigma-Aldrich) for 20 minutes, and was related to whole membrane protein as previously described [44].

### Formation of steroid glucuronides

LNCaP shADRB2 and shCtrl protein lysates were prepared by centrifugation at 890g for 10 min at 4°C followed by lysis by sonication on ice. The lysates were diluted in PBS supplied with 0.5 mM dithiothreitol (DTT) (GE Healthcare, Buckinghamshire, UK). Xenograft homogenates were prepared by homogenizing 50 μg of tumor tissues from mice injected with LNCaP shADRB2 and shCtrl in 250 μL of ice-cold PBS supplied with 0.5 mM DTT using a Homogenizer Motor Drives (Glass-Col Homogenizer #099C-K54, Terre-Haute, IN, USA). Steroid glucuronidation assays were performed by adding 10 μL of lysates (8.8 μg/μL) or xenograft homogenates (5 μg/μL) to a glucuronidation assay buffer (50 mM Tris-HCl pH 7.5, 10 mM MgCl2, 10μg/mL phosphatidylcholine, 1 mM uridine 5′-diphosphoglucuronic acid (UDPGA), 2.5 μg/mL pepstatin, 0.5 μg/mL leupeptin, 0.025 μg/mL alamethicin, and 100 μM of either testosterone, DHT, 3α-Diol, or androsterone dissolved in ethanol). The mixtures were incubated at 37°C for 1 or 4 hours (cell lysates or xenograft homogenates, respectively) before quenching the glucuronidation reactions with 2 nM ice-cold methanol:butylated hydroxytoluene (BHT). Proteins were centrifuged at 13,000 rpm for 10 minutes at 4°C to pellet the protein precipitate. Supernatants were used for glucuronide quantification by LC-MS/MS as previously reported [47]. Cell lysate samples used for glucuronidation assays were prepared with the same dilutions of those used for immunoblotting assays, so the protein levels directly correspond to the glucuronidation activity. HEK293-cells and ethanol were used as negative controls, and a pool of human liver samples as positive control.

### Androgen quantification

Testosterone levels in cell cultures was measured by a multi-steroid LC-MS/MS assay as described by [48] with the following modifications: Isotope-labeled internal standards (10 μl) and 190 μl 50 % acetonitrile was added to 85 μl of each calibrator and quality control as well as to each cell pellet lysate. The samples were sonicated (40% amplitude, 10 pulses of 1s), equilibrated at RT for one hour, and extracted by liquid-liquid extraction with 850 μl ethylacetate:hexane (80:20). After centrifugation, 650 μl of the organic phase was dried under a stream of N_2_ for 30 minutes at 40°C. The samples were reconstituted in 50 μl 25% methanol, and 10 μl of each sample was injected into a Waters Xevo TQ-S that was coupled to an i-class Acquity UPLC. The chromatographic system consisted of a 100 × 2.1 mm Acquity BEH C-18 column (1.8 μM particle size) heated to 60° C, and two mobile phases consisting of Milli-Q purified water with 0.05% ammonium hydroxide (A) and methanol with 0.05% ammonium hydroxide (B). Samples were separated by a linear gradient from 30% to 75% mobile phase B over 7.5 min at a flow-rate of 0.4 ml/min. Testosterone was quantitated by electrospray ionisation in positive mode, and multiple reaction monitoring as described previously [48]. Concentrations were calculated as fmol per cell pellet or tumor tissue weight.

### Statistics

Time to development of CRPC and time to prostate cancer-specific mortality was analyzed by competing risk regression using the Fine and Grey model [49], while Cox proportional hazards modelling was applied for all-cause mortality analysis. The proportional hazards assumption was assessed by a test based on Schoenfeld residuals. There was no evidence of violation of the assumption in any model (all *p*-values > 0.13). Correlations were calculated using Spearman's rank correlation, due to non-normality of the data. For all other statistical analyses, a two-tailed Student's t-test or Fischer exact test were used to determine statistical significance. For xenograft tumor glucuronide formation, values were log_10_-transformed to invoke normally distributed data prior to parametric testing. Statistics were performed using SPSS version 18, STATA version 12 and Microsoft Excel. A two-sided *p*-value of < 0.05 was considered statistically significant for all analyses.

## SUPPLEMENTARY INFORMATION TABLES AND FIGURES


