# Supplementary material for: Low β2-adrenergic receptor level may promote development of castration resistant prostate cancer and altered steroid metabolism
Source: Oncotarget. 2015 Dec 4;7(2):1878–94. doi: 10.18632/oncotarget.6479 (PMC4811504; doi:10.18632/oncotarget.6479)
Supplement: Supplementary file 1 [file oncotarget-07-1878-s001.pdf]

Low β_2-adrenergic receptor level may promote development of castration resistant prostate cancer and altered steroid metabolism

Supplementary Information

MATERIAL AND METHODS

ADRB2 antibody specificity and sensitivity

Multiple antibodies targeting ADRB2 were tested on western blots, and the antibody chosen for the immunohistochemical staining recognized both the correct band from an ADRB2 over-expression lysate from HEK293T-cells (LY424968, Origene, Rockville, MD) and only one single band from patient tissue protein extracts which' size corresponds to the theoretical size. The antibody only bound luminal cells, which is in compliance with prior immunohistochemical analyses using other antibodies in prostate tissue. Finally, immunofluoresence of shADRB2 cells showed reduced signal intensity (data not shown).

Supplementary Table 1: List of plasmid names, vectors, comments and the source/reference of the plasmids.

Plasmid name	Vector	Comment/insert	Source/reference
shCtrl	SureSilencing shRNA plasmid	GGAATCTCATTCGATGCATAC	Qiagen (Cat no. KH01856N)
shADRB2-1		TGAGACCTGCTGTGACTTCTT	Qiagen (Cat no. KH01856N)
shADRB2-2		GGCAACTTCTGGTGCGAGTTT	Qiagen (Cat no. KH01856N)
pCDNA3.1-ADRB2	pCDNA3.1	Constitutively expresses ADRB2 with a Flag tag protein	Tang et al. 1999
pEGFP-C3	pEGFP-C3	Constitutively expresses GFP under CMV-promoter	Clontech
pPB(-285/132)-Luc	pGL3	Gift from F. Saatcioglu (University of Oslo, Norway)	Palvimo et al. 1996
285-Pb-pEZX-PG04	pEZX-PG04	pPb(-285/132)-Luc was Cloned into Gluc-ON^{TM} Promoter clone system	GeneCopoeia
pEZX-PG04	pEZX-PG04		GeneCopoeia
pGL3/PSA	pGL3	Gift from S. Balk (Dana-Farber/Harvard Cancer Center, MA)	Oettgen et al. 2000

Androgen receptor (AR) western blots

The LNCaP shADRB2 and shCtrl cells were harvested, lysed in whole cell buffer, and centrifuged at 16,000 g for 20 min. Immuno-blots were prepared and visualized as described in materials and methods. Anti-AR (1:1000, Santa Cruz Biotechnology, TX, USA) was used as primary antibody, with α-tubulin (1:1000, Sigma-Aldrich) as loading control.

RESULTS

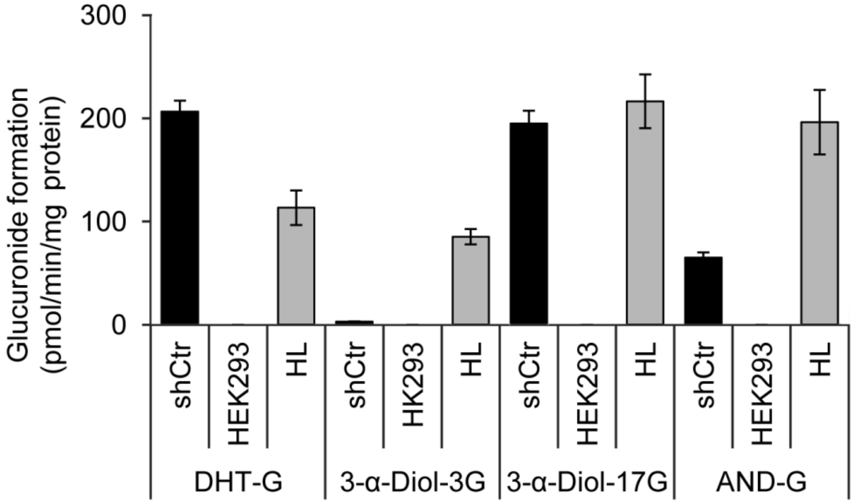

Supplementary Figure 1: Formation of androgen glucuronides in positive and negative controls. Homogenates from HEK293 cells (negative control) and human liver samples (HL, positive control) were mixed with UDPGA and either dihydrotestosterone (DHT), 3α-androstanediol (3α-Diol) or androsterone (ADT) for one hour, and levels of conjugated androgens were measured by LC-MS/MS. The results are shown as mean formed glucuronide related to total protein in the homogenates (pmol/min/mg protein) from duplicated reactions on three biological replications ± SEM. The values obtained with LNCaP shCtrl are shown to compare glucuronidation rates with the controls.

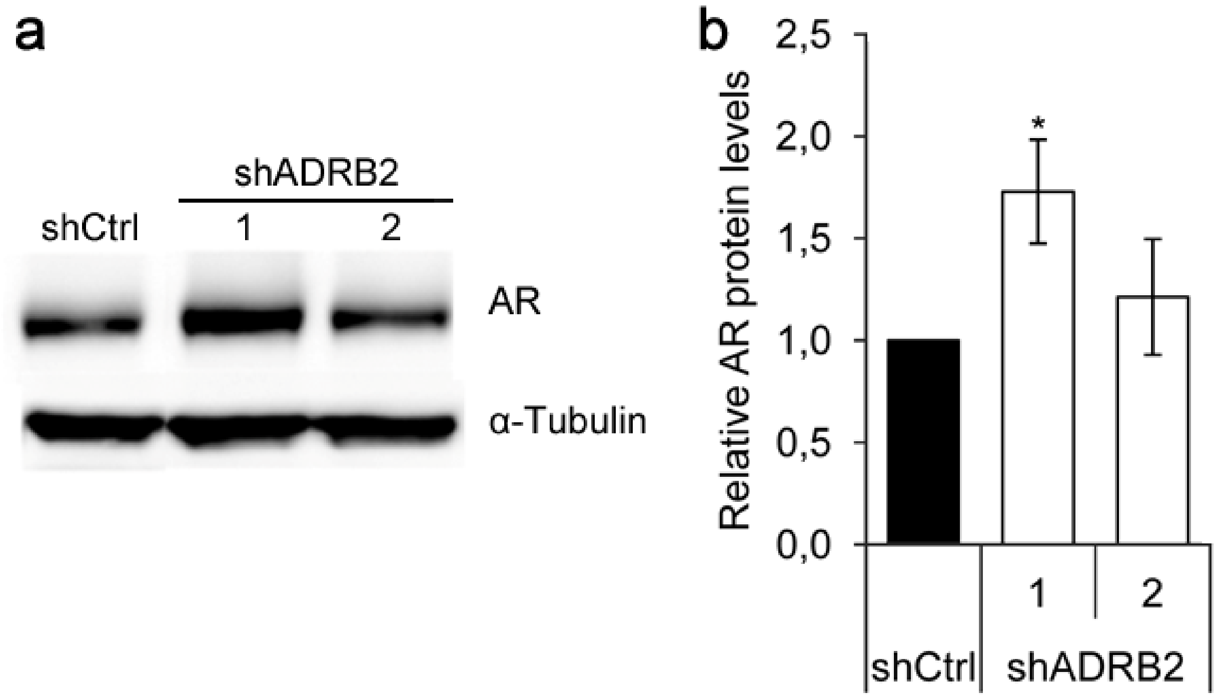

Supplementary Figure 2: Androgen receptor (AR) protein level. (a) The AR protein level was visualized in cell protein extracts by immunoblotting using an anti-AR antibody (1:1000). An anti-tubulin antibody (1:1000) was simultaneously used on the same extracts to ensure similar loading on the lanes. **(b)** Mean, relative AR protein intensities from three independent western blot experiments are shown ± SD.

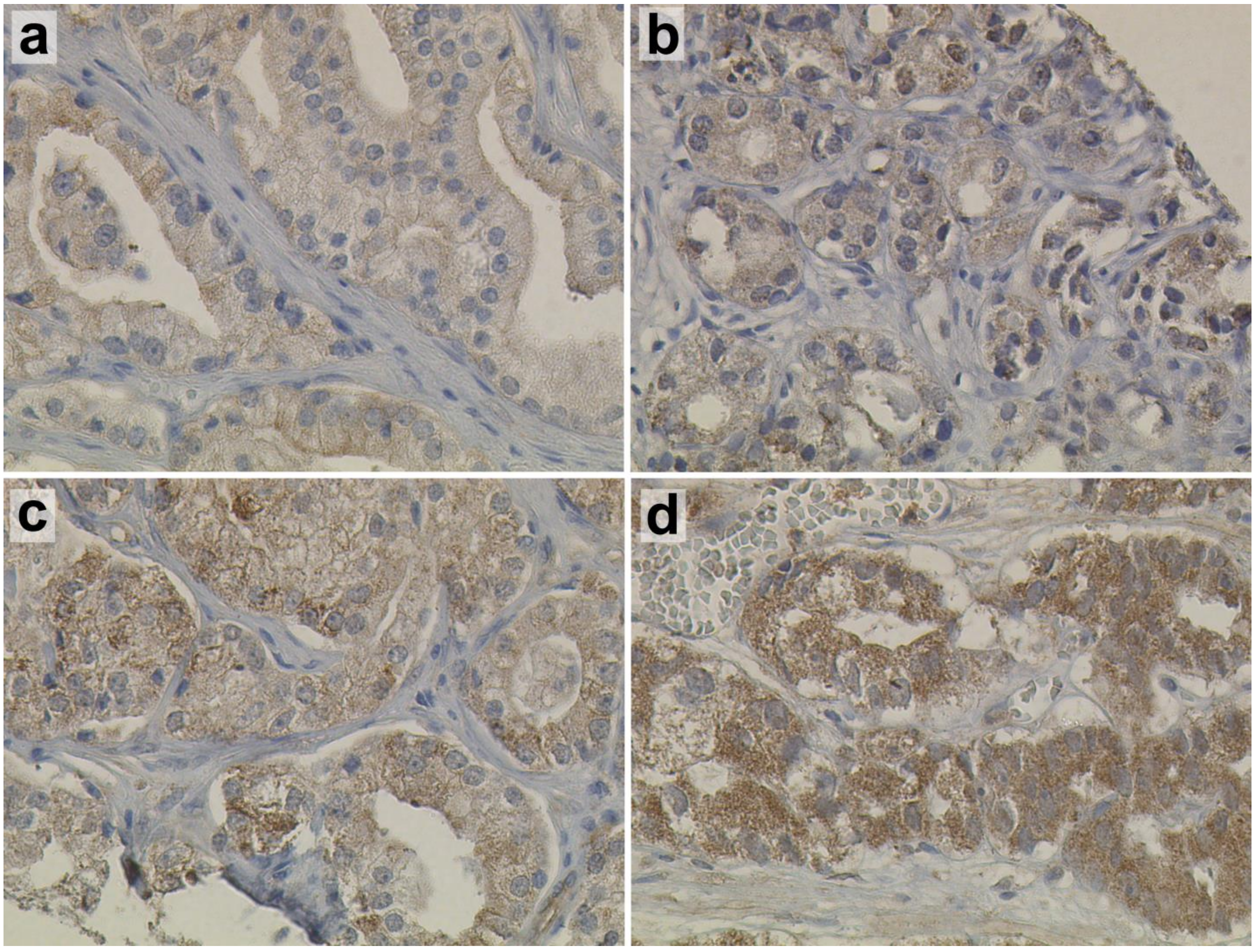

Supplementary Figure 3: Immunohistochemical analysis of UGT2B15 and UGT2B17 expression in the Oslo ADT TMA of transurethral resections of the prostate (TUR-P). Examples of tissue cores of Gleason 7b showing **(a)** weak UGT2B15, **(b)** intermediate UGT2B15, **(c)** weak UGT2B17**,** or **(d)** intermediate UGT2B17 staining are presented at 400X magnification.

Supplementary Table 2: Clinical characteristics at diagnosis for patients included in the immunohistochemical analyses of ADRB2.

**Clinical characteristic at diagnosis**	**Number of patients (n=40)**
Gleason score	
2-6 or highly differentiated	13
7 or intermediately differentiated	13
8-10 or poorly differentiated	14
Unknown	0
Clinical T-stage	
1-2	5
3-4	14
Unknown	21
PSA-level	
< 4.0	0
4.1-10.0	3
10.1-20.0	5
> 20.0	19
Unknown	13
Metastasis	
Yes	3
No	20
Unknown	17
Age at diagnosis (mean (SD))	69.3 (8.3)
